# Simultaneous processing and degradation of mitochondrial RNAs revealed by circularized RNA sequencing

**DOI:** 10.1093/nar/gkx104

**Published:** 2017-02-15

**Authors:** Irina Kuznetsova, Stefan J. Siira, Anne-Marie J. Shearwood, Judith A. Ermer, Aleksandra Filipovska, Oliver Rackham

**Affiliations:** 1 Harry Perkins Institute of Medical Research and Centre for Medical Research, The University of Western Australia, Nedlands 6009, Australia; 2 School of Molecular Sciences, The University of Western Australia, Crawley 6009, Australia

## Abstract

Mammalian mitochondrial RNAs are unique as they are derived from primary transcripts that encompass almost the entire mitochondrial genome. This necessitates extensive processing to release the individual mRNAs, rRNAs and tRNAs required for gene expression. Recent studies have revealed many of the proteins required for mitochondrial RNA processing, however the rapid turnover of precursor RNAs has made it impossible to analyze their composition and the hierarchy of processing. Here, we find that circularization of RNA prior to deep sequencing enables the discovery and characterization of unprocessed RNAs. Using this approach, we identify the most stable processing intermediates and the presence of intermediate processing products that are partially degraded and polyadenylated. Analysis of libraries constructed using RNA from mice lacking the nuclease subunit of the mitochondrial RNase P reveals the identities of stalled processing intermediates, their order of cleavage, and confirms the importance of RNase P in generating mature mitochondrial RNAs. Using RNA circularization prior to library preparation should provide a generally useful approach to studying RNA processing in many different biological systems.

## INTRODUCTION

In the pathway from gene to protein, many different forms of regulation have been identified that can control the final amounts of individual proteins that are produced. In prokaryotes, regulation occurs predominantly at the level of transcription, while in eukaryotes post-transcriptional regulation is more prevalent, however this can vary greatly from organism to organism. In some unusual systems, a lack of regulation at the transcriptional level has necessitated the evolution of mechanisms to potently modulate the production of different proteins post-transcriptionally. For example, in kinetoplastida RNA polymerase II is almost completely unregulated and produces vast swathes of RNA often encompassing hundreds of kilobases of genomic sequence and many different genes (1). In order to have the correct ratios of these different proteins kinetoplastida use a sophisticated network of RNA-binding proteins to alter the stabilities of each different mRNA, which is now quite well understood (2). An analogous situation occurs in mammalian mitochondria, where both strands of the mitochondrial genome are transcribed as two long polycistronic RNAs encompassing the whole genome sequence (3,4). However, in this case, we are only now beginning to understand the mechanisms controlling the levels of individual mitochondrial proteins (4–6).

When the sequence of the mitochondrial genome was first elucidated it was observed that the genes encoding individual mitochondrial rRNAs and mRNAs were almost always separated by genes for tRNAs (7). This led to the speculation that cleavage of these tRNAs from the precursor RNA transcripts would enable the production of a full set of individual RNAs required for mitochondrial gene expression (known as the ‘tRNA punctuation model’) (8). Cleavage of mitochondrial tRNAs is performed by a protein-only RNase P (consisting of mitochondrial RNase P proteins 1, 2 and 3; encoded by the *MRPP1, MRPP2* and *MRPP3* genes) at their 5΄ ends and the mitochondrial RNase Z (encoded by the *ELAC2* gene) at their 3΄ ends (9–11). The cleavage of tRNAs from the long precursor RNAs has been shown to be important for the production of mature tRNAs and also mRNAs (10,11), rRNAs and non-coding RNAs, and consequently RNA maturation, ribosome assembly and protein synthesis (11,12). To better understand the importance of RNA processing in mitochondrial gene expression we have previously combined knockdown or knockout of components of the RNA processing machinery with RNA sequencing (RNA-Seq) and parallel analysis of RNA ends (PARE) (11–13). These approaches have revealed much regarding the roles of different proteins in these processes but can be limited by the short read lengths produced by current deep sequencing platforms. Because of this feature current approaches to analyze RNA by deep sequencing incorporate an RNA fragmentation step to produce sequences of a manageable length, however this eliminates information on the exact length and composition of the original longer RNAs. To circumvent this limitation we circularized individual RNA molecules prior to library construction to preserve the identities of their 5΄ and 3΄ termini in the final sequencing data. Circularization of RNA followed by deep sequencing has been used to study mutations in viral RNAs (‘CirSeq’) (14) and for capture of low abundance small RNA fragments (‘RC-Seq’) (15) but has not previously been used to study RNA processing. Using this approach we identify rare processing intermediates in normal mitochondria and stalled intermediates produced when mitochondrial RNase P function is lost.

## MATERIALS AND METHODS

### Animals and housing

*Mrpp3* transgenic mice on a C57BL/6N background were generated by Taconic (Cologne, Germany). Heart- and skeletal muscle-specific knockout mice were generated by crossing *Mrpp3^loxP/loxP^* mice with transgenic mice expressing Cre under the control of the muscle creatinine kinase promoter (*Ckmm-cre*). Double heterozygous mice (*Mrpp3^loxP/+^, +/Ckmm-cre*) were mated with *Mrpp3^loxP/loxP^* mice to generate heart-specific knockout (*Mrpp3^loxP/loxP^, +/Ckmm-cre*) and control mice (*Mrpp3^loxP/loxP^*). Mice were housed in standard cages (45 cm × 29 cm × 12 cm) under a 12-h light/dark schedule (lights on 7 am to 7 pm) in controlled environmental conditions of 22 ± 2°C and 50 + 10% relative humidity and fed a normal chow diet (Rat & Mouse Chow, Specialty Foods, Glen Forrest, Western Australia) and water were provided *ad libitum*. The study was approved by the Animal Ethics Committee of the UWA and performed in accordance with Principles of Laboratory Care (NHMRC Australian code for the care and use of animals for scientific purposes, 8th Edition 2013).

### Mitochondrial isolation

Mitochondria were isolated from homogenized hearts and purified by differential centrifugation as described previously (12,16) with some modifications. Hearts were cut and washed three times with ice cold PBS, and once with mitochondrial isolation buffer (MIB) containing 310 mM sucrose, 10 mM Tris–HCl and 0.05% BSA (w/v) by centrifugation at 4500 *g* for 1 min at 4°C. Heart pieces were homogenized in 5 ml of fresh MIB using a Potter S pestle. The homogenate was centrifuged at 1000 *g* for 10 min at 4°C and the supernatant was centrifuged at 4500 *g* for 15 min at 4°C to isolate mitochondria.

### RNA isolation and northern blotting

RNA was isolated from total hearts or heart mitochondria using the miRNeasy Mini kit (Qiagen) incorporating an on-column RNase-free DNase digestion to remove all DNA. For studies of the *mt-Co3* mRNA, Terminator 5΄-phosphate-dependent exonuclease digestions were performed using 2.5 μg of RNA as recommended by the manufacturer (Epicentre), using either a high activity reaction buffer (Reaction Buffer A) or a high specificity reaction buffer (Reaction Buffer B). For northern blotting, RNA (8 μg) was resolved on 1.2% agarose formaldehyde gels, then transferred to 0.45 μm Hybond-N^+^ nitrocellulose membrane (GE Lifesciences) and hybridized with biotinylated oligonucleotide probes specific to mouse mitochondrial RNAs. Hybridizations were carried out overnight at 50°C in 5× SSC, 20 mM Na_2_HPO_4_, 7% SDS and 100 μg.ml^−1^ heparin, followed by washing. The signal was detected using either streptavidin-linked horseradish peroxidase or streptavidin-linked infrared-labelled antibody (diluted 1: 2000 in 3× SSC, 5% SDS, 25 mM Na_2_HPO_4_, pH 7.5) by enhanced chemiluminescence (GE Lifesciences) or using an Odyssey Infrared Imaging System (Li-Cor), respectively.

### Circularized RNA library preparation

Libraries were prepared based on the methods of Chu *et al*. (15). RNA circularization was performed with 5 or 25 U/μl CircLigase II ssDNA Ligase (Epicentre) in 1× reaction buffer (Epicentre) with 2.5 mM MnCl_2_ and 1 M Betaine at 60°C for 1 h. To remove any remaining linear RNA an RNase R digestion was performed at 37°C for 10 min (1 U/μl, Epicentre). After the digestion, the circularized RNA was purified using Oligo Clean & Concentrator columns following the manufacturer's instructions (Zymo). RNA was reverse transcribed with the following primer: 5΄-GACGTGTGCTCTTCCGATCTNNNNNN-3΄, using Superscript II according to the manufacturer's instructions (Invitrogen, Thermo Fisher Scientific). RNA was hydrolyzed with 0.1 M KOH and heating at 95°C for 15 min, then reactions were cooled to room temperature and neutralized by adding HCl to 0.1 M. The cDNA was purified with DNA Clean & Concentrator columns and extended with Klenow DNA polymerase I (NEB) using the following primer: 5΄-ACACGACGCTCTTCCGATCTNNNNNNNN-Phos-3΄, in Buffer 2 (NEB) with 4 mM DTT at 25°C for 15 min, and then at 95°C for 3 min to deactivate the enzyme. The tagged cDNA was purified with DNA Clean & Concentrator columns (Zymo) and amplified using the Failsafe PCR system (Premix E, Epicentre) using the following forward primer: 5΄-AATGATACGGCGACCACCGAGATCTACACTCTTTCCCTACACGACGCTCTTCCGATCT-3΄, and one of the following reverse primers (Illumina, indexed sequence is shown in bold font): 5΄-CAAGCAGAAGACGGCATACGAGAT**CGTGAT**GTGACTGGAGTTCAGACGTGTGCTCTTCCGATCT-3΄, 5΄-CAAGCAGAAGACGGCATACGAGAT**ACATCG**GTGACTGGAGTTCAGACGTGTGCTCTTCCGATCT-3΄, 5΄-CAAGCAGAAGACGGCATACGAGAT**GCCTAA**GTGACTGGAGTTCAGACGTGTGCTCTTCCGATCT-3΄, 5΄-CAAGCAGAAGACGGCATACGAGAT**TGGTCA**GTGACTGGAGTTCAGACGTGTGCTCTTCCGATCT-3΄, 5΄-CAAGCAGAAGACGGCATACGAGAT**CACTGT**GTGACTGGAGTTCAGACGTGTGCTCTTCCGATCT-3΄, or 5΄-CAAGCAGAAGACGGCATACGAGAT**ATTGGC**GTGACTGGAGTTCAGACGTGTGCTCTTCCGATCT-3΄. Reactions were heated at 95°C for 1 min, followed by 18 cycles of 95°C for 30 s, 55°C for 30 s and 68°C for 3 min with a final extension step at 68°C for 7 min. PCR products were purified using Agencourt AMPure XP magnetic beads at a 1:1 volume ratio. Library size distribution was analyzed by D1000 ScreenTape analysis (Agilent) and then quantitated by qPCR.

### Sequencing and primary data processing

Sequencing of each library was performed on an Illumina MiSeq using standard Illumina sequencing primers: HP10 read 1, HP12 i7 index seq primer, HP11 read 2. Sequencing was performed to generate 250 bp paired-end reads and 0.5% of a PhiX control library (Illumina) was spiked in for the sequencing run. Adapter trimming was performed using cutadapt software (17) with minimum length set to 20 nt and the trimmed pair-end reads were merged by FLASH (18). The minimum overlapping length between two reads was left as default (10 nt), the maximum set to 245 nt. The program output was three files, where one of the files contained reads that were combined into one long sequence (merged reads), and the remaining two files contained the first and second reads that were not merged (unmerged reads). A file that contained merged reads and a file that contained the first read that was not merged by FLASH was used for further analysis for each of the replicates (18). Next, we identified repetitive sequences within reads by applying Tandem Repeats Finder (TRF) software to all files (19). The TRF output file was converted from dat file format to fastq format.

### Genome alignment

Alignment to the mouse mitochondrial genome was performed using bowtie2 (20). The soft-clipping mode was used for the alignment, the remaining unaligned parts were extracted based on the CIGAR field information from the alignment (21). As smaller RNAs, such as tRNAs, are present in multiple tandem repeats, due to a rolling circle mode of reverse transcription of circularized RNAs, we often observe more than two copies of them. For convenience, the split parts of each read are called ‘left’, ‘middle’ and ‘right’. Retrieved ‘left’ and ‘right’ parts were aligned again to the mouse mitochondrial genome, but this time using an end-to-end mode (20). Once all these steps were completed for merged and unmerged reads, the final aligned datasets, including replicates, were combined for analysis. The generated files for the *Mrpp3* knock outs (‘KO’) and matched wild-type (‘WT’) control ‘left’, ‘middle’, ‘right’ were used for further analysis. Coverage was visualized using the Integrative Genomics Viewer (IGV) (22,23).

### Mapping of precursor RNA ends

The merged file was used to identify intermediate processing product parts. Because split parts of reads that come from the same read share the same ID, split-mapping reads can be identified. Therefore, if part of a read mapped to another part of the genome it can be represented as a link and visualized using Circos software (24). For each link the middle part of the read was used to build a line that is connected to the right or left part of the read. Thereby finding the start and end of each intermediate processing product. In order to calculate the log fold change (log2FC) the coverage of combined data was found by BEDtools genomecov (25) and normalized to total reads mapped to the mitochondrial genome (counts per million; CPM). Intermediate steps were achieved using Python v2.7.6 (retrieved from https://docs.python.org/2.7/) and R v3.3.1 (retrieved from https://www.R-project.org/).

### Bioinformatic analyses of small RNAs

Small RNAs within the circularized RNA sequencing libraries were extracted from the repetitive sequences within reads identified by Tandem Repeats Finder (TRF) (19). For comparison small RNA sequencing libraries were constructed and sequenced from wild-type and *Mrpp3* knockout heart mitochondria using standard Illumina methodologies. Sequenced reads were trimmed of adapters with cutadapt (17) with a minimum length of 15 nt. Trimmed reads were aligned with bowtie2 (20) to the mouse reference genome sequence (mm10) which had been masked for NUMT regions, as defined by the UCSC mm9 numtS track with coordinates converted to mm10 with UCSC liftOver. Alignments were filtered to remove true multireads (MAPQ > = 2) and coverage was calculated with BEDtools (25), normalized to total reads mapped to chrM. Cutadapt was used to identify reads with 3΄-CCA (-a CCA$ -e 0.0 -O 3 -n 1) or poly(A) (-a A{20} -e 0.0 -O 3 -n 1) sequences with a length of at least 3, and their unique read IDs were extracted. The alignments were filtered with BEDtools intersect to extract reads that overlap tRNA regions by at least 80%, allowing for 3΄ extensions (-s -f 0.8), or reads that overlap rRNA and mRNA regions entirely, excluding true 3΄ poly(A) tails (-s -f 1.0). The tRNA-, rRNA- and mRNA-overlapping alignments were filtered with Picard tools using the -CCA and -polyA read ID lists to extract tRNA-aligned reads with 3΄ -CCA ends, and rRNA- and mRNA-aligned polyadenylated reads, excluding the canonical 3΄ poly(A) tail. The coverage of total and 3΄-CCA tRNA-aligned reads at the 3΄ terminal nucleotide was computed in each sample and the ratio of the two calculated. The coverage of total and poly(A) rRNA- and mRNA-aligned reads was calculated similarly.

### Rapid amplification of cDNA ends (RACE)

The 5΄ ends of RNA extracted from wild-type and *Mrpp3* knockout hearts were captured using the RLM-RACE kit (Ambion, Thermo Fisher Scientific), according to the manufacturer's recommendations, except the calf intestine alkaline phosphatase and tobacco acid pyrophosphatase treatments were omitted since mitochondrial RNAs are not capped. The following gene-specific primers were used to identify the 5΄ ends of *mt-Co1*: 5΄-GTACCCACTATTCCCGCTCA-3΄, 5΄-TATTCCCGCTCAGGCTCCGAATAG-3΄. The 3΄ ends of specific RNAs were captured using the Targeted RNA Tagging RT-PCR Assay of Lapointe *et al*. (26), except the Terminator nuclease treatment was omitted, since mitochondrial RNAs are not capped, and a universal or poly(A)-specific anchor primers were used in place of the ‘U-select primer’, since no poly(U) polymerase was present in our samples. The following gene-specific primers were used to identify the 3΄ ends of intermediate processing products containing *mt-Co1*: 5΄- CACATTCGAGGAACCAACCT-3΄, 5΄-GGTTTCAAGCCAATCTCATATCCTATATG-3΄.

## RESULTS

### Circularized RNA sequencing of mitochondrial RNAs

**Figure 1.**
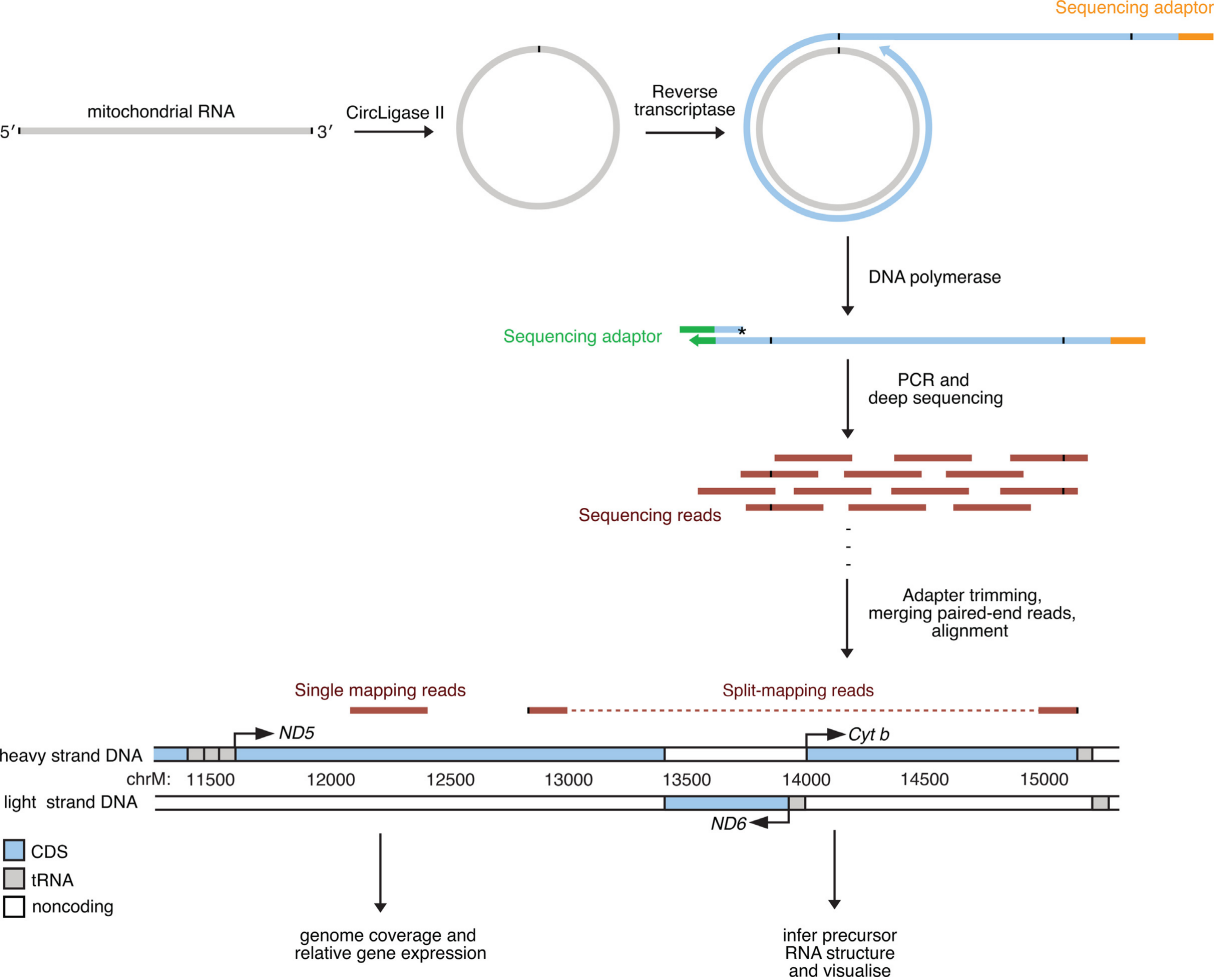
Schematic illustrating the preparation and analysis of circularized RNA libraries. Purified RNA was treated with a thermostable single-stranded nucleic acid-specific ligase (CircLigase II) to achieve intramolecular ligation of individual molecules. After ligation any remaining linear RNA was degraded using RNase R and the circular RNAs were reverse transcribed using random primers incorporating an adaptor sequence for subsequent sequencing. A second adaptor sequence was added by extension of the cDNA using a 3΄-end-blocked oligonucleotide as a template. The cDNA was then amplified by limited PCR. Primers were designed to incorporate sequences for subsequent deep sequencing.

To bypass the problems associated with current RNA-Seq technologies we took inspiration from the RNA library preparation methods of Chu *et al*. (15) and Acevedo *et al*. (14), where RNA is circularized prior to reverse transcription. The intramolecular ligation of RNA is much more efficient than using small oligonucleotide adaptors (15). Furthermore, for studying the composition of long RNAs this provides a unique advantage as the 5΄ and 3΄ ends of each individual RNA are joined together, preserving this information in the subsequent deep sequencing data. We isolated mitochondria from mouse hearts using subcellular fractionation by differential centrifugation, to enrich for mitochondrial RNAs. Intramolecular ligation of purified RNA was achieved using a thermostable single-stranded nucleic acid-specific ligase (CircLigase II). Because of its thermostability, ligation reactions could be performed at 60°C to reduce the chances of RNA secondary structure biasing the ligation efficiencies of different RNAs. Conveniently, CircLigase II only catalyzes the ligation of nucleic acids bearing a 5΄-phosphate and a 3΄-hydroxyl group, which is thought to be a characteristic of all mammalian mitochondrial RNAs (4,27). After ligation any remaining linear RNA was degraded using RNase R and the circular RNAs were reverse transcribed using random primers. The cDNA was then made double stranded using Klenow DNA polymerase and amplified by limited PCR. Primers were designed to incorporate sequences for Illumina sequencing primers enabling their subsequent deep sequencing (Figure 1).

### Computational analyses of circularized RNA sequencing libraries

Because circularized RNAs are reverse transcribed by rolling circle amplification sequencing reads can include direct repeats of shorter RNA sequences. Therefore, we identified repeated sequences within reads and collapsed them to a single parental sequence, if required (15,19). In addition, because circularized RNAs could give rise to reads that bridge the 5΄ and 3΄ ends of RNAs we used a soft-clipping and end-to end mode approach to align reads to the mouse mitochondrial genome (20). We kept the best aligning matches for each region of the read and their alignment positions to enable subsequent analysis of single and split-mapping reads.

### Library optimization and analyses for potential biases

**Figure 2.**
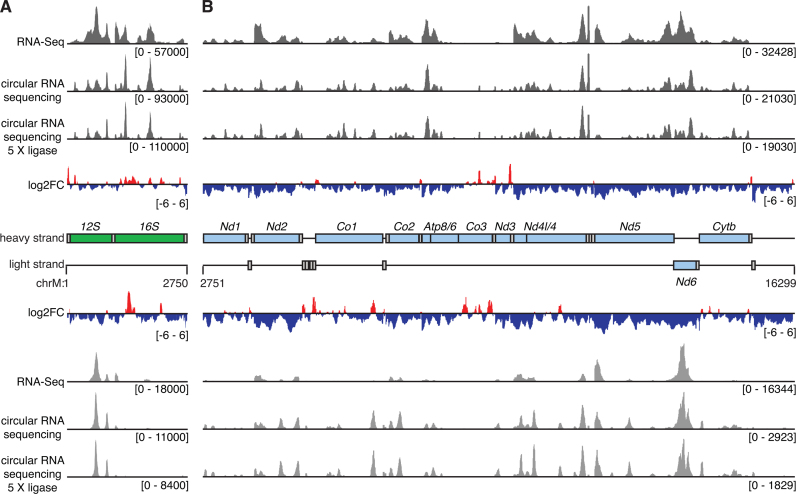
Analysis of coverage from libraries produced by circularized RNA sequencing compared to standard RNA-Seq. The mitochondrial genome is separated into the region containing the rRNAs (**A**) and mRNAs (**B**). Coverage is shown in dark grey for heavy strand-encoded RNAs and in light gray for light strand-encoded transcripts. The ratio of coverage from circularized RNA sequencing compared to RNA-Seq is shown as log_2_ fold change with regions of increased coverage in red and decreased coverage in blue. The locations of genes are shown in a central schematic, where genes encoding rRNAs are shown in green, mRNAs are shown in light blue and tRNAs in gray. The ‘mt-’ prefix is omitted from each gene name for clarity.

The circularization of input RNA might introduce potential biases into the final sequencing libraries, for example this reaction could in principle favor the circularization of smaller RNAs. To examine this phenomenon we produced a library where the input RNA was ligated with a higher concentration of CircLigase II. We found that this variation did not alter the distribution of reads covering the mitochondrial genome, indicating that a ligation bias based on the length of input RNAs did not significantly alter the libraries produced in this approach (Figure 2), however we note that based on biophysical principles that ligation of longer RNAs is likely to be less efficient and might reduce the coverage of very long RNAs. Furthermore, we did not examine the effect of the adaptor sequences incorporated in our adaptors that were necessary for subsequent deep sequencing. Nevertheless, previous work by Chu *et al*. (15) showed that there was no sequence bias inherent in the ligation by CircLigase II. Importantly, comparison of the coverage produced using circularized RNA sequencing with RNA-Seq data (12) showed that there was more uniform coverage in circularized RNA sequencing, with better representation of regions of the mitochondrial genome that include rRNAs and tRNAs (Figure 2).

### Identification of partially processed mitochondrial RNAs

**Figure 3.**
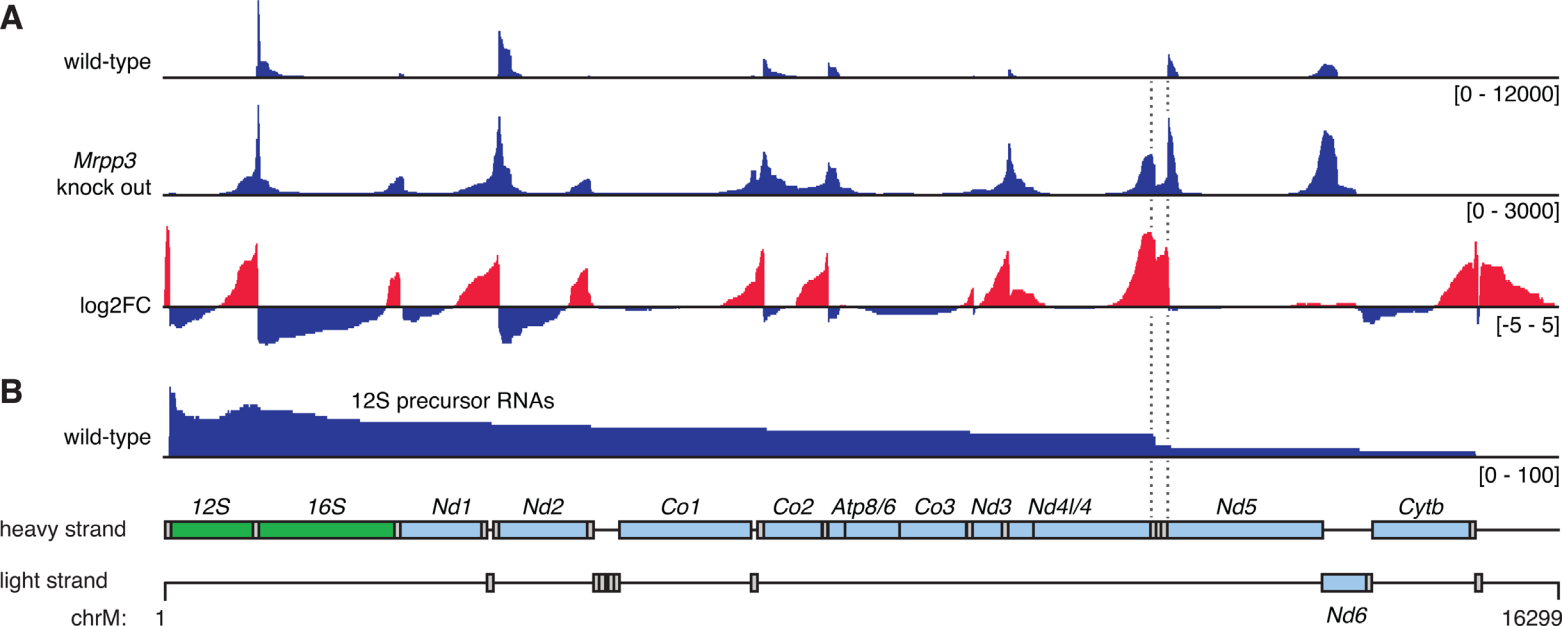
Analysis of partially processed transcripts in libraries produced by circularized RNA sequencing. Abundance of reads which map between multiple genes are shown across the mitochondrial genome in both wild-type mouse mitochondria and *Mrpp3* knock out mitochondria (**A**). A log_2_ fold change (log_2_FC) highlights partially processed regions that are enriched in the absence of MRPP3 with regions of increased coverage in red and decreased coverage in blue. Partially processed transcripts containing the 12S rRNA are shown in (**B**) and the region containing *mt-Th, mt-Ts2* and *mt-Tl2*, is highlighted by dotted lines. The locations of genes are shown in a schematic, where genes encoding rRNAs are shown in green, mRNAs are shown in light blue and tRNAs in gray.

**Figure 4.**
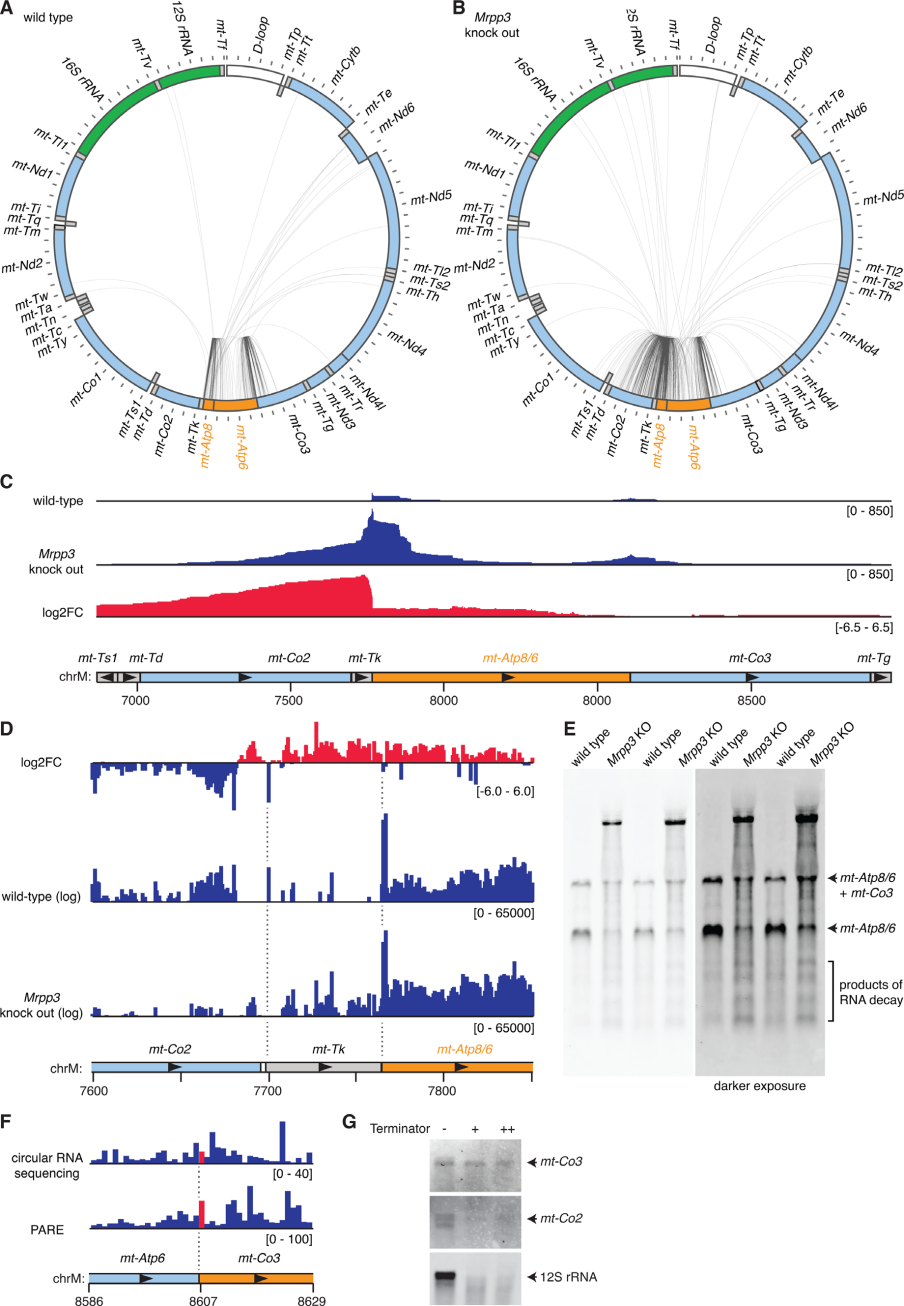
Analysis of partially processed transcripts containing *mt-Atp8/6*. (**A**) Circularized RNA sequencing of wild-type mouse mitochondrial RNA. Reads mapping between *mt-Atp8/6* and other mitochondrial RNAs are illustrated by lines between the 5΄ and 3΄ nucleotide positions. The locations of genes are shown in a schematic, where heavy strand genes are shown in the outer ring and light strand genes in the inner ring. Genes encoding rRNAs are shown in green, mRNAs are shown in light blue, tRNAs in gray, the non-coding D-loop in white, and the mRNA of interest, *mt-Atp8/6*, is shown in orange. (**B**) Circularized RNA sequencing of mitochondrial RNA from *Mrpp3* knock out mice. (**C**) Circularized RNA sequencing data illustrating the increase in coverage of unprocessed transcripts containing *mt-Atp8/6* in the absence of MRPP3. (**D**) PARE data illustrating the reduction in 5΄ and 3΄ processing of *mt-Tk* in the absence of MRPP3. (**E**) Northern blotting of heart RNA from wild-type and *Mrpp3* knock out mice using a probe specific for *mt-Atp8/6*. (**F**) The 5΄ ends of reads mapping with the genomic region containing the 5΄ end of *mt-Co3* are shown from the circularized RNA sequencing and PARE data. The putative 5΄ end position of *mt-Co3* is highlighted in red in the data and the *mt-Co3* mRNA is highlighted in orange in the schematic. (**G**) A 5΄-phosphate-dependent exonuclease (terminator exonuclease) was used to digest isolated mouse mitochondrial RNA using either a high specificity reaction buffer (lane indicated by a +) or a high activity reaction buffer (lane indicated by ++). Specific RNAs were subsequently detected by northern blotting.

**Figure 5.**
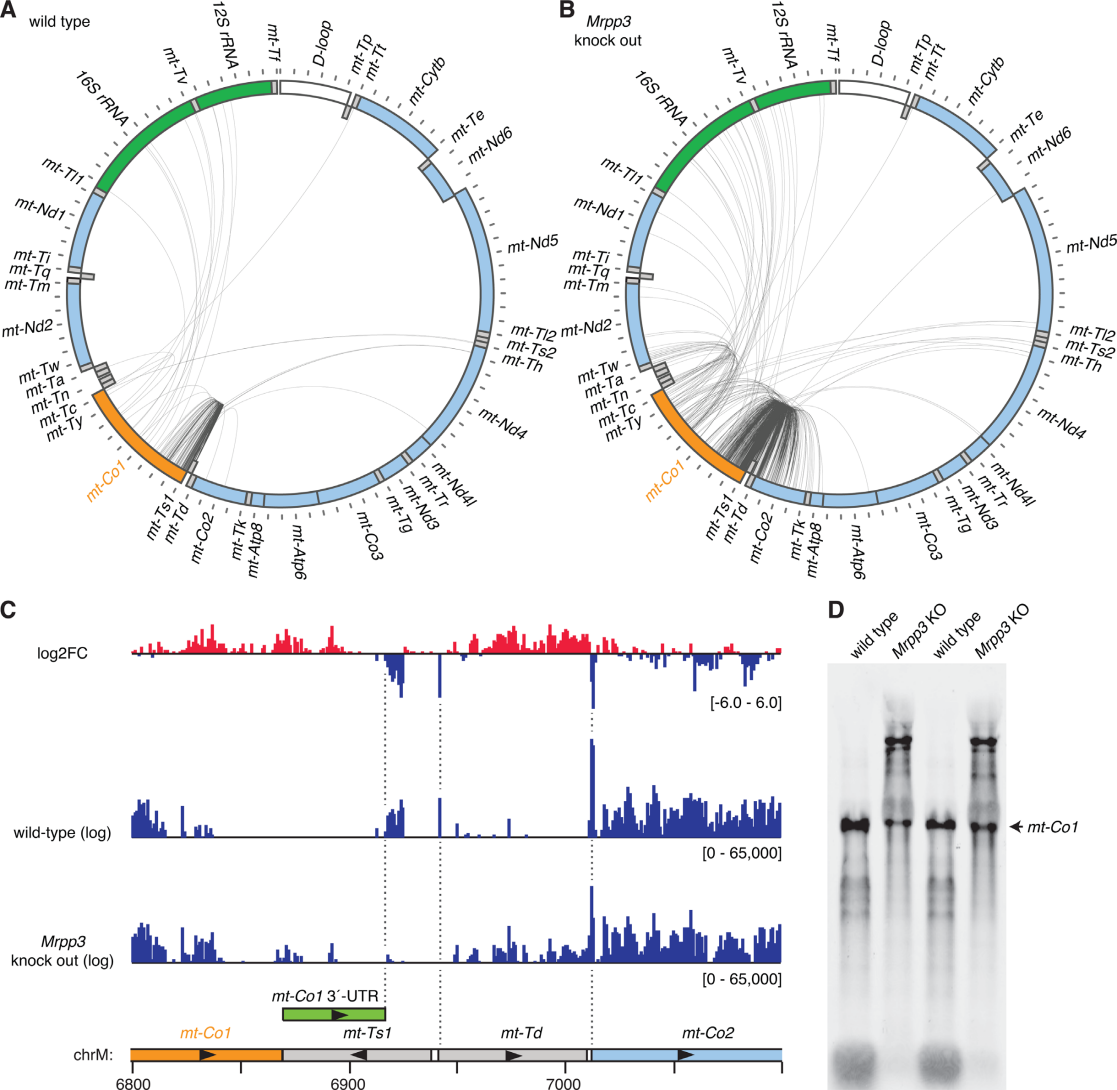
Analysis of partially processed transcripts containing *mt-Co1*. (**A**) Circularized RNA sequencing of wild-type mouse mitochondrial RNA. Reads mapping between *mt-Co1* and other mitochondrial RNAs are illustrated by lines between the 5΄ and 3΄ nucleotide positions. The locations of genes are shown in a schematic, where heavy strand genes are shown in the outer ring and light strand genes in the inner ring. Genes encoding rRNAs are shown in green, mRNAs are shown in light blue, tRNAs in grey, the D-loop in white, and the mRNA of interest, *mt-Co1*, is shown in orange. (**B**) Circularized RNA sequencing of mitochondrial RNA from *Mrpp3* knock out mice. (**C**) PARE data illustrating the reduction in 5΄ and 3΄ processing of *mt-Td* in the absence of MRPP3 and also the position of *mt-Co1*'s 3΄-UTR (12). (**D**) Northern blotting of heart RNA from wild-type and *Mrpp3* knock out mice using a probe specific for *mt-Co1*.

**Figure 6.**
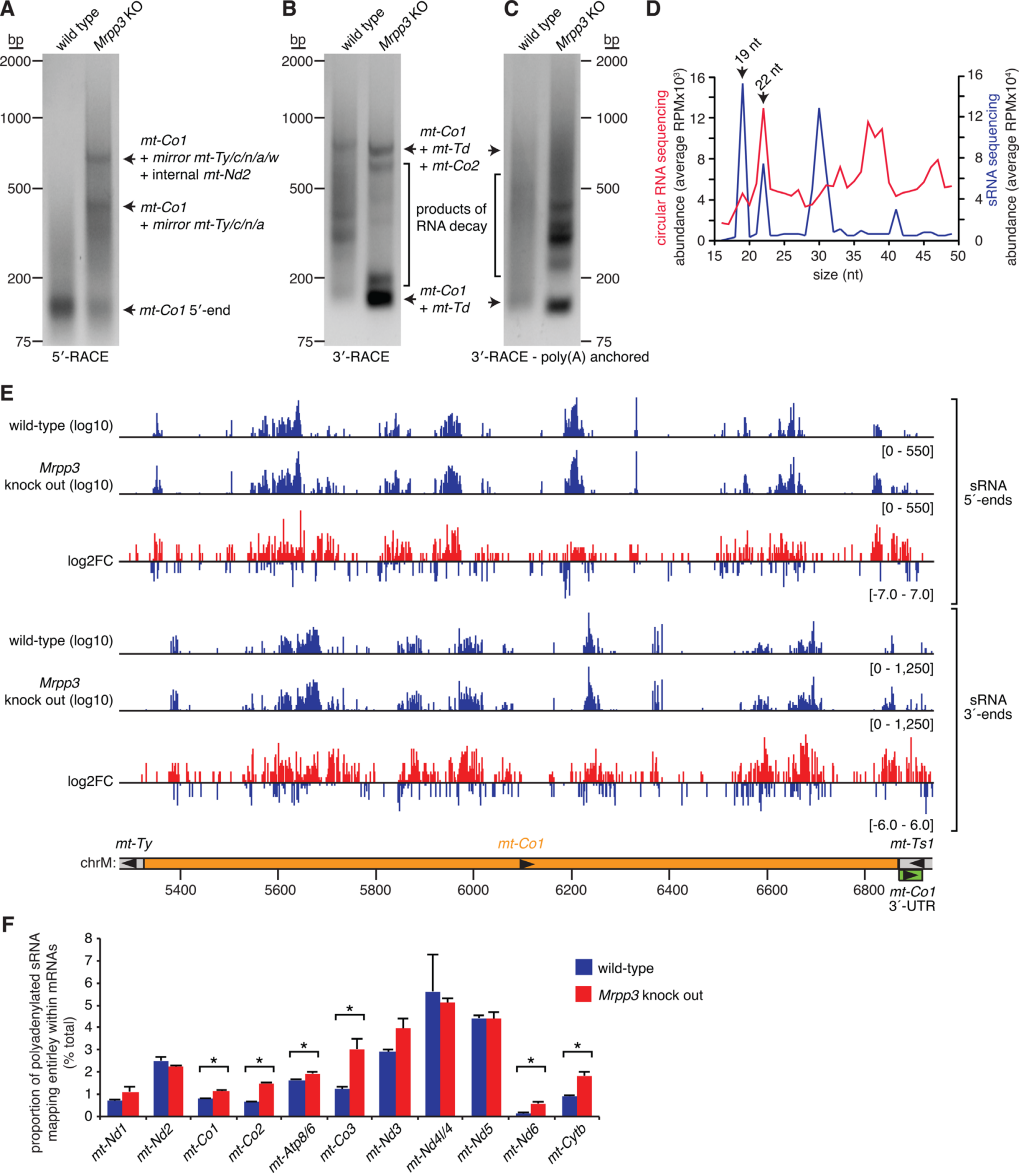
Polyadenylation of mRNA decay intermediates. (**A**) 5΄ rapid amplification of cDNA ends (5΄-RACE) validates the 5΄ ends of *mt-Co1*. 3΄-RACE identified total (**B**) and polyadenylated (**C**) processing intermediates that include *mt-Co1*. Circularized RNA sequencing captured authentic small RNAs (**D**). The abundance of small RNAs (sRNAs) of different sizes identified by circularized RNA sequencing were compared to those detected by classical sRNA sequencing. The positions of the 19 and 22 nt sRNAs previously identified in the human mitochondrial transcriptome (27) are indicated by arrows. (**E**) Locations of sRNA 5΄ and 3΄ ends within *mt-Co1* and the fold change between wild-type and *Mrpp3* knockout mitochondrial circularized RNA libraries are shown. Small RNAs derived from *mt-Co1* were increased in abundance in the *Mrpp3* RNA libraries (Student's *t*-test, *P* = 0.0034). (**F**) A proportion of sRNA degradation products derived from mRNAs are polyadenylated. The proportion of polyadenylated sRNAs derived from the following mRNAs were significantly increased upon loss of MRPP3 (Student's *t*-test, *P* ≤ 0.05, indicated by an asterisk): *mt-Co1, mt-Co2, mt-Atp8/6, mt-Co3, mt-Nd6* and *mt-Cytb*.

**Figure 7.**
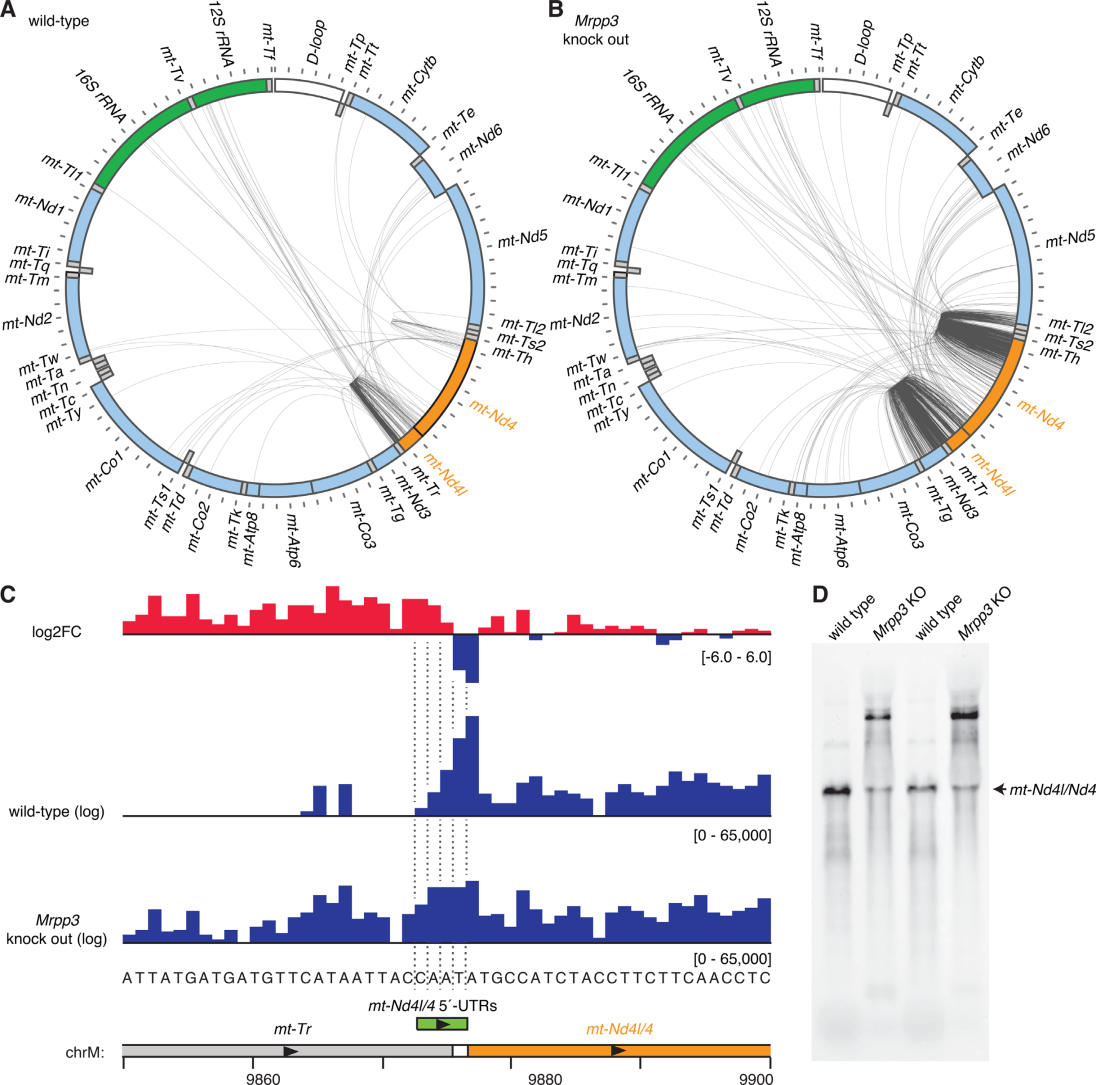
Analysis of partially processed transcripts containing *mt-N4l/4*. (**A**) Circularized RNA sequencing of wild-type mouse mitochondrial RNA. Reads mapping between *mt-Nd4l/4* and other mitochondrial RNAs are illustrated by lines between the 5΄ and 3΄ nucleotide positions. The locations of genes are shown in a schematic, where heavy strand genes are shown in the outer ring and light strand genes in the inner ring. Genes encoding rRNAs are shown in green, mRNAs are shown in light blue, tRNAs in grey, the D-loop in white, and the mRNA of interest, *mt-Nd4l/4*, is shown in orange. (**B**) Circularized RNA sequencing of mitochondrial RNA from *Mrpp3* knock out mice. (**C**) PARE data illustrating the various positions of *mt-Nd4l/4*'s short 5΄-UTRs. (**D**) Northern blotting of heart RNA from wild-type and *Mrpp3* knock out mice using a probe specific for *mt-Nd4l/4*.

We used split-mapping reads from our circularized RNA sequencing libraries to discover the ends of intermediate processing products from mitochondrial RNAs in normal mouse heart mitochondria. These represented 1.7% of the total mitochondrial RNA sequences, however this likely underrepresents the abundance of these transcripts as many single mapping reads may have been produced from unprocessed RNAs or intermediate processing products where the reads did not bridge their 5΄ and 3΄ ends. Visualization of the distribution of these RNAs revealed the most frequently occurring processing intermediates (Figure 3A). In wild-type mitochondria we found very few intermediate processing products extending through the tRNA cluster that includes *mt-Th, mt-Ts2* and *mt-Tl2*, indicating that cleavage at these tRNAs occurs most rapidly. Focusing on partially processed reads that include the 12S rRNA, frequent cleavage at the *mt-Th/mt-Ts2/mt-Tl2* cluster was also readily apparent (Figure 3B). To examine the most prevalent partially processed forms of individual RNAs of interest we generated maps illustrating the positions of reads mapping within these RNAs and at another location in the mitochondrial genome (Figures 4–7). These maps reveal the intermediates that occur in the generation of these transcripts. The distribution of precursors was highly reproducible between libraries made from three independent mice (Supplementary Figure S1). The predominant transcript ends were typically idiosyncratic to individual RNAs, and specific examples are discussed below.

### Accumulation of partially processed mitochondrial RNAs in the absence of MRPP3

We have previously shown that the tissue-specific deletion of the *Mrpp3* gene in mice, encoding one of the three mitochondrial RNase P subunits, results in a severe RNA processing defect and accumulation of RNAs representing intermediate processing products (12), however the exact composition of these RNAs was unknown. We produced circularized RNA sequencing libraries from mitochondria isolated from three independent *Mrpp3* knockout hearts and interrogated them for partially processed transcripts using the analysis methods described above. The number of intermediate RNA processing products in libraries made from *Mrpp3* knock out mitochondria was double that detected in wild-type mitochondria. The most significant changes were observed in regions of RNA containing tRNAs (Figure 3A). Comparison with our previous data on RNase P processing sites, generated using parallel analyses of RNA ends (PARE) (12) showed good agreement in the sites that were most affected by loss of MRPP3, such as *mt-Tf, mt-Tv* and the *mt-Th/mt-Ts2/mt-Tl2* cluster. Examination of the maps of RNA processing intermediates revealed the identities of stalled processing intermediates and sites that require RNase P for their processing, and specific examples are discussed below.

### Partially processed transcripts containing *mt-Atp8/6*

The *mt-Atp8/6* transcript is one of two bicistronic mRNAs found in mammalian mitochondria. This transcript is flanked at its 5΄ end by *mt-Tk*, encoding tRNA^Lys^, however it is unusual because its 3΄ end lacks a tRNA but is nevertheless cleaved before the following mRNA *mt-Co3*. In wild-type mice we observed reads that mapped to both *mt-Atp8/6* and *mt-Co3*, indicating that the noncanonical cleavage between these RNAs is inefficient compared to processing between *mt-Tk* and *mt-Atp8/6*. A long-lived *mt-Atp8/6-mt-Co3* transcript, has been observed previously in humans (28) and we can also detect this transcript by northern blotting of wild-type mouse RNA (Figure 4E), confirming the inefficient cleavage between *mt-Atp8/6* and *mt-Co3* RNAs. In wild-type mice we did not observe any intermediate RNA processing products mapping between *mt-Atp8/6* and *mt-Co2* (Figure 4A), however in MRPP3 knockout mice 30% of partially processed RNAs mapped to *mt-Co2* (Figure 4B). This was confirmed by examining the coverage of partially processed transcripts and the fold change in coverage between wild-type and *Mrpp3* knock out libraries (Figure 4C). Cleavage of the 3΄ ends of mitochondrial tRNAs often requires prior cleavage at their 5΄ ends. Examination of previously published parallel analyses of RNA ends (PARE) from *Mrpp3* knock out mice shows that loss of MRPP3 results in complete loss of processing at the 5΄ end of *mt-Tk*, however cleavage at its 3΄ end is only mildly reduced (Figure 4D). Therefore, defective cleavage at the 5΄ end of *mt-Atp8/6* results in the accumulation of the partially processed RNAs observed by northern blotting in the absence of MRPP3 (Figure 4E).

Interestingly we observed that many 5΄ and 3΄ ends fell within the *mt-Co2* and *mt-Co3* open reading frames, indicating that these were not only processing intermediates but also decay intermediates. Longer exposures of northern blots enabled the visualization of partially degraded *mt-Atp8/6-*derived transcripts (Figure 4E, right panel), verifying these observations from our deep sequencing data. In libraries constructed from *Mrpp3* knockout heart mitochondria we observed an increase in transcript ends mapping within the *mt-Co2* and *mt-Co3* open reading frames, indicating enhanced degradation, which was also confirmed by northern blotting (Figure 4E). This confirms our previous observations that the compensatory increase in transcription in *Mrpp3* knockout heart mitochondria results in increased degradation of RNA. Indeed northern blotting showed an increase in 7S RNA (Supplementary Figure S2), which is a marker of transcription from mtDNA, in our *Mrpp3* knock out samples.

### 5΄ ends of *mt-Co3*

In our circularized RNA sequencing data we did not see any significant mapping of 5΄ read ends to the 5΄ end of *mt-Co3* (Figure 4F). This was also observed in PARE data, where no 5΄ ends were captured beyond the local background. Although both circularized RNA sequencing and PARE capture RNAs to be sequenced via ligation of the 5΄ transcript ends, they use very different ligases, reaction conditions, and PARE uses an intermolecular ligation, while circularized RNA sequencing proceeds via intramolecular ligation. Since both circularized RNA sequencing and PARE require a 5΄ monophosphate for the initial ligation step, we performed 5΄-phosphate-dependent exonuclease digestion of isolated mouse mitochondrial RNA to examine if a particular biochemical feature of the *mt-Co3* mRNA was responsible for its absence in the different deep sequencing libraries. Northern blotting of digested RNA revealed that the *mt-Co3* mRNA was not susceptible to 5΄-phosphate-dependent exonuclease digestion, unlike the *mt-Co2* and 12S rRNA transcripts (Figure 4G). This suggests that the *mt-Co3* mRNA lacks a 5΄ monophosphate or that its 5΄ monophosphate is inaccessible for exonuclease digestion due to the RNA's secondary structure. Given that our circularized RNA sequencing libraries were prepared via ligation at 60°C to disrupt RNA secondary structure, we favor the former hypothesis.

### Partially processed transcripts containing *mt-Co1*

The *mt-Co1* gene is a heavy strand encoded ORF flanked on both sides by light strand tRNAs (*mt-Ty* at its 5΄ end and *mt-Ts1* at its 3΄ end). In wild-type mouse RNA we found very few reads indicative of intermediate RNA processing products (Figure 5A). The majority of reads mapped to position 6,917, which is 45 nt after the *mt-Co1* stop codon. Based on previous studies (29) and our PARE data (Figure 5C) this likely constitutes the 3΄-UTR of this transcript, which is unusual as it is one of only three mitochondrial mRNAs with significant 3΄-UTRs, along with *mt-Nd5* and *mt-Nd6* (28,30). Therefore circularized RNA sequencing can also be useful for determining the true ends of RNAs. The number of reads mapping to position 6917 was significantly decreased in circularized RNA sequencing from knock out mice, indicating that the RNase P complex is required to generate the *mt-Co1* 3΄-UTR (Figure 5B). This is corroborated in the PARE *Mrpp3* dataset (12), where fewer 5΄ ends were mapped immediately downstream of this location (Figure 5C). The *mt-Co1* 3΄-UTR is derived from RNA that is encoded by the strand complementary to that encoding *mt-Ts1*. These RNA regions, dubbed ‘mirror tRNAs’, often fold into tRNA-like structures. We used computational prediction of the structure of the RNA in this region and show that it is highly reminiscent of a tRNA (Supplementary Figure S3), with one stem that is very similar to the acceptor stem of mitochondrial tRNA^Val^, providing additional evidence that it might be recognized directly by RNase P. However, it is also possible that an additional nuclease could cleave at this site, and RNase P cleavage at distal sites might simply facilitate its access to this RNA.

In *Mrpp3* knockout heart mitochondria, there were dramatically more reads mapping outside of the *mt-Co1* gene (Figure 5B). Precursor RNAs mapped to both 5΄ and 3΄ tRNAs and mRNAs (*mt-Nd2, mt-Tw* and *mt-Td, mt-Co2*, respectively), however more mapped to the 3΄ side, likely because the four consecutive light strand tRNA genes, folding as ‘mirror tRNAs’, at the 5΄ side provide many potential ELAC2 cleavage sites between *mt-Nd2* and *mt-Co1* (12). The variety of potential ELAC2 cleavage sites on either side of the *mt-Co1* mRNA are reflected in the highly heterogeneous partially processed RNAs observed in northern blots probing for this RNA in *Mrpp3* knockout samples (Figure 5D).

### Polyadenylation of partially processed transcripts and small RNA products of RNA degradation

To provide additional information on the RNA species detected by circularized RNA sequencing we performed rapid amplification of cDNA ends (RACE) for the 5΄ and 3΄ ends of *mt-Co1*. We observed that partially processed RNAs extending 5΄ of *mt-Co1* were not readily detected in wild-type mitochondria, however in the absence of RNase P activity products mapping to the canonical 5΄ end of *mt-Co1* were reduced and two predominant extensions corresponding to precursors cleaved at the 3΄ end of *mt-Tw* and a site internal to *mt-Nd2* were apparent (Figure 6A). 3΄-RACE detected a predominant processing intermediate that included *mt-Co1* and *mt-Td* in the absence of RNase P, as well as products extending to the 3΄ end of *mt-Co2* and sites internal to *mt-Co2* (Figure 6B). These partially processed ends were also the major RNA ends detected in analyses of *mt-Co1* processing intermediates using circularized RNA sequencing. These data illustrate the concordance between the circularized RNA sequencing data and identities of intermediate processing products found in mitochondrial RNA samples by molecular biology assays.

When performing the 3΄-RACE assays described above we also included samples processed using a 3΄-RACE anchor primer that specifically amplifies polyadenylated transcripts and observed that many partially processed RNAs that were concurrently degraded, as they were cleaved internal to *mt-Co2*, were polyadenylated (Figure 6C). To examine this in more detail, we took advantage of the fact that circularized RNA sequencing also captures small RNAs (sRNAs) to measure RNA decay products. Small RNAs are reverse transcribed by rolling circle amplification and form tandem repeats in the resulting deep sequencing. We extracted these using tandem repeat deconvoluting software (19) and compared them to sRNAs sequenced from matched samples using conventional sRNA-Seq. The sRNAs detected by both methods included the common 19 and 22 nt species we had previously discovered in human cells (27), validating that circularized RNA sequencing can be used to study authentic sRNAs (Figure 6D). We examined sRNAs derived from mRNAs in our libraries that would result from the degradation of these mRNAs. These RNAs were scattered throughout the mitochondrial mRNAs and often increased in abundance in the *Mrpp3* RNA libraries (Figure 6E, Supplementary Figure S4). These sRNAs likely correspond to authentic RNA decay intermediates as they show good concordance with abundant RNA degradation intermediates identified by 3΄-RACE (Supplementary Figure S5). Interestingly, we found substantial polyadenylation of these mRNA degradation products (Figure 6F). Furthermore, we note that the proportion of these polyadenylated degradation products increased in our *Mrpp3* knockouts, where we see increased RNA degradation, and was statistically significant for *mt-Co1, mt-Co2, mt-Atp8/6, mt-Co3, mt-Nd6*, and *mt-Cytb* mRNAs.

### Unprocessed transcripts containing *mt-Nd1*

The *mt-Nd1* gene is representative of the typical structure of mammalian mitochondrial mRNAs, in that it is flanked by tRNA genes on either side (*mt-Tl1* at its 5΄ end and *mt-Ti* at its 3΄ end). In wild-type mitochondrial RNA we detected very few unprocessed RNAs containing *mt-Nd1* (Supplementary Figure S6A), although most mapped to its 5΄ flank confirming previous observations that a longer lived intermediate, known as RNA19, that includes *mt-Nd1*, tRNA^Leu1^ and the 16S rRNA is found in mitochondria. We confirmed that the RNA19 intermediate was present in our samples by northern blotting (Supplementary Figure S6D).

In *Mrpp3* knockout heart mitochondria there was an increase in partially processed RNAs mapping to either side of *mt-Nd1* (Supplementary Figure S6B, including *mt-Tl1*, 12S rRNA at its 5΄ flank and *mt-Ti*, mirror *mt-Tq, mt-Tm* and *mt-Nd2* at the 3΄ side). Cleavage of both the 5΄ and 3΄ ends of *mt-Tl1* are lost in *Mrpp3* knockout mice, while only cleavage of the 3΄ end of *mt-Ti* is not impaired in the *mt-Ti/mt-Tq/mt-Tm* tRNA cluster (Supplementary Figure S6C) (12). Therefore it is not surprising that large partially processed RNAs are observed in the absence of RNase P, by both circularized RNA sequencing (Supplementary Figure S6B) and northern blotting (Supplementary Figure S6D).

### Unprocessed transcripts containing *mt-Nd4l/4*

The *mt-Nd4l/4* gene is the second of the two bicistronic mRNAs and is flanked at its 5΄ end by *mt-Tr*, and at its 3΄ end by the *mt-Th/mt-Ts2/mt-Tl2* tRNA cluster. Our circularized RNA sequencing of wild-type mitochondrial RNA revealed sparse mapping of partially processed RNAs at either flank of *mt-Nd4l/4* (Figure 7A), with the exception of a large number of reads mapping just one, two or three nucleotides upstream of the *mt-Nd4l/4* ATG start codon. This confirms previous observations that *mt-Nd4l/4* has a very short 5΄-UTR (29) and examination of PARE data showed the same trend as circularized RNA sequencing where 1 nt was the most frequent 5΄-UTR length, followed by 2, 3 and then very rarely 4 nt (Figure 7C). Production of 5΄-UTRs longer than 1 nt requires nucleotides from the 3΄ end of tRNA^Arg^ (encoded by *mt-Tr*) to be included in the *mt-Nd4l/4* transcript. Interestingly the final two mtDNA-encoded nucleotides of tRNA^Arg^ are both adenines, raising the possibility that productive tRNAs could still be generated from tRNA^Arg^ cleaved at these positions via repair by poly(A) polymerase, as has been observed recently for tRNA^Tyr^ after removal of tRNA^Cys^ from the tRNA^Tyr^–tRNA^Cys^ precursor RNA (31).

Production of *mt-Nd4l/4*'s short 5΄-UTR ends requires RNase P activity for their production, as reads capturing these sites were significantly reduced in circularized RNA sequencing and PARE from *Mrpp3* knockout heart mitochondria (Figure 7B and C). Whether RNase P directly cleaves these sites or if they are cleaved by another enzyme, such as ELAC2, that can only function after RNase P processing of upstream sites remains to be determined. Indeed, in the absence of RNase P activity circularized RNA sequencing produced a large number of reads mapping between *mt-Nd4l/4* and its flanking mRNA genes (Figure 7B and C), including between *mt-Nd4l/4* and *mt-Nd5*, despite the fact that three tRNAs separate these mRNAs. This confirms previous findings that loss of mitochondrial RNase P can impact the processing of tRNAs that likely represent ELAC2 substrates (12). Interestingly, we also found that the proportion of tRNA-derived reads bearing the post-transcriptional 3΄CCA addition was decreased in our deep sequencing of small RNAs (Supplementary Figure S7), indicating that reduced cleavage at the 5΄ ends of tRNAs can not only impair ELAC2 cleavage at the 3΄ end of tRNAs but also the subsequent addition of the CCA trinucleotide.

## DISCUSSION

In summary, we used deep sequencing of circularized RNA to discover the composition of longer-lived mitochondrial RNA precursors both in normal heart mitochondria and in mitochondria lacking proteinaceous RNase P activity. In circularized RNA sequencing from mitochondria lacking MRPP3, and hence RNase P activity, we found a dramatic increase in reads bridging multiple genes. This revealed the most sensitive sites to loss of RNase P and allowed the imputation of the structures of partially processed RNAs. Many precursor RNAs we observed included multiple tRNAs, that in principle contain ELAC2 cleavage sites. This confirms previous findings that RNase P processing of tRNAs is obligatory for subsequent RNase Z/ELAC2 cleavage in most cases in mammalian mitochondria (12). Furthermore, the impact on tRNA 3΄end maturation goes beyond cleavage as the addition of the CCA trinucleotide, that is essential for aminoacylation of tRNAs, was reduced in mitochondria lacking MRPP3. These observations confirm the interdependent nature of the pathways required to produce mature RNAs within mitochondria (12,32).

Interestingly, we observed partially processed RNAs that were cleaved within open reading frames of downstream or upstream RNAs, indicating that processing and degradation can occur simultaneously within mitochondria. Whether these represent defective RNAs destined for complete degradation or whether there is some ‘collateral damage’ of individual portions of the long precursor RNAs during their maturation remains to be determined. Nevertheless, these results reconcile observations of short RNA half-lives in mitochondria (33) and apparent long half-lives of mitoribosomes (containing ‘short-lived’ rRNAs) (32,34) since a proportion of rRNAs are likely rapidly degraded before processing is complete, while those that are assembled correctly into mitoribosomes are stabilized for a long period of time.

The process of RNA degradation in mammalian mitochondria is still poorly understood. A complex of the SUV3 helicase and the polynucleotide phosphorylase (PNPase) is required but its substrate preferences and regulation have not been established to date. Expression of a dominant negative form of the SUV3 helicase in tissue cultures cells resulted in the production of a large proportion of truncated transcripts (between 36% and 57% of RNAs) (35). Interestingly, truncated transcripts were also detected in control cells (between 1.5% and 15% of RNAs) (35), supporting our observations that partially degraded transcripts are often present at significant amounts. Furthermore, SUV3 was found to be important for removal of processing intermediates (35). Our observations of partially degraded processing intermediates provide further evidence that degradation of mitochondrial RNAs can occur concomitantly with processing.

Although the mitochondrial genome is densely packed with genes, because both strands are transcribed in their entirety there is significant non-coding RNA produced that constitutes the antisense of mRNAs and tRNAs (4). Although we have previously shown that some of these long non-coding RNAs are abundant, particularly those from the *Nd5-Cytb* region (30), there remains a need to dispose of the majority of these non-coding RNAs. How the degradation system in mitochondria differentiates between canonical mRNAs, rRNAs and tRNAs and intervening RNAs destined for destruction remains to be determined. Polyadenylation is traditionally known as an mRNA stabilizing mechanism in cytoplasm and mitochondria of eukaryotes. In mammals the activity of the mitochondrial poly(A) polymerase (MTPAP) is enhanced by the LRPPRC/SLIRP complex, whose loss reduces both mRNA stability and polyadenylation (29,33). However in plant and kinetoplastida mitochondria polyadenylation can be involved in promoting RNA degradation (36,37), and in a recent study loss of MTPAP in *Drosophila* did not impact RNA stability but instead resulted in impaired protection of the 3΄ ends of certain mRNAs and tRNAs, as well as CCA addition to a subset of tRNAs (38). In this study we observed degradation intermediates, including both long partially processed RNAs and small RNA fragments that are polyadenylated. Furthermore, we observed increased polyadenylation of RNA decay products upon deletion of *Mrpp3*, indicating that polyadenylation may also play an important role in mRNA decay in mammalian mitochondria. Finally, we also measured a reduction in CCA addition to the 3΄ ends of tRNAs upon loss of MRPP3, illustrating the complex relationship between RNA maturation and decay pathways in mammalian mitochondria.

The ability of circularized RNA sequencing to detect rare unprocessed RNAs could have broad applicability in studies of RNA metabolism. The methods used in this study specifically capture RNAs with 5΄-phosphate and 3΄-hydroxyl groups but could be easily modified to sequence RNAs with alternative terminal structures. This is particularly relevant to subsets of nuclear-encoded transcripts, where treatments with decapping enzymes and phosphatases would be beneficial. In the case of mitochondria this might only be relevant to the two 5΄ ends that are generated from the initiating nucleotide triphosphates of the heavy and light precursor transcripts, and also the 5΄ end of *mt-Co3* that we identified in this work as being resistant to ligation by CircLigase II and also cleavage by a 5΄-phosphate-dependent exonuclease. It is possible that the FASTKD5 protein may play a role in the processing of this site, since knockdown of this protein results in an increased accumulation of the *mt-Atp8/6-mt-Co3* transcript (39).

Preparation of libraries by intramolecular circularization of RNA can facilitate the sequencing of samples where amounts of RNA are limiting or where the structures of individual RNAs prevent intermolecular ligation (15). This enables rare RNAs to be analyzed with better coverage, allowing more accurate characterization of processing intermediates and degradation products without the requirement for chemical or genetic disruption of the proteins involved in these processes. This could be useful to reveal previously undiscovered aspects of the lifecycles of RNAs.

## ACCESSION NUMBERS

The accession numbers for the RNA sequencing data reported in this paper are Gene Expression Omnibus (GEO): GSE94005 and GSE94030.

## Supplementary Material

Supplementary Data
