# Supplementary material for: Simultaneous processing and degradation of mitochondrial RNAs revealed by circularized RNA sequencing
Source: Nucleic Acids Res. 2017 Feb 15;45(9):5487–500. doi: 10.1093/nar/gkx104 (PMC5435911; doi:10.1093/nar/gkx104)
Supplement: Supplementary Data [file gkx104_Supp.pdf]

Supplementary Material for:

Simultaneous processing and degradation of mitochondrial RNAs revealed by circularized RNA sequencing

Irina Kuznetsova[a], Stefan J. Siira[a], Anne-Marie J. Shearwood[a], Judith A. Ermer[a], Aleksandra Filipovska[a,b], Oliver Rackham[a,b1]

[a] Harry Perkins Institute of Medical Research and Centre for Medical Research, The University of Western Australia, Nedlands, Australia.

[b] School of Chemistry and Biochemistry, The University of Western Australia, Crawley, Australia.

[1] To whom correspondence should be addressed.

This file contains:

Supplementary Figure 1. Reproducibility of circularized RNA sequencing libraries and analyses.

Supplementary Figure 2. Levels of the 7S RNA are increased in *Mrpp3* knock out mice.

Supplementary Figure 3. The predicted secondary structure for the *mt-Co1* 3′-UTR RNA.

Supplementary Figure 4. Locations of sRNA 5′ and 3′ ends within *mt-Co1* and the fold change between wild-type and *Mrpp3* knockout mitochondrial circularized RNA libraries, upon normalization to reads mapping to the whole genome.

Supplementary Figure 5. Correspondence between small RNA degradation products in *mt-Co2* and partially processed transcripts containing *mt-Co1* that extend into this region identified by 3′-RACE.

Supplementary Figure 6. Analysis of partially processed transcripts containing *mt-Nd1*.

Supplementary Figure 7. Analysis of CCA addition to the 3′ ends of mitochondrial tRNAs.

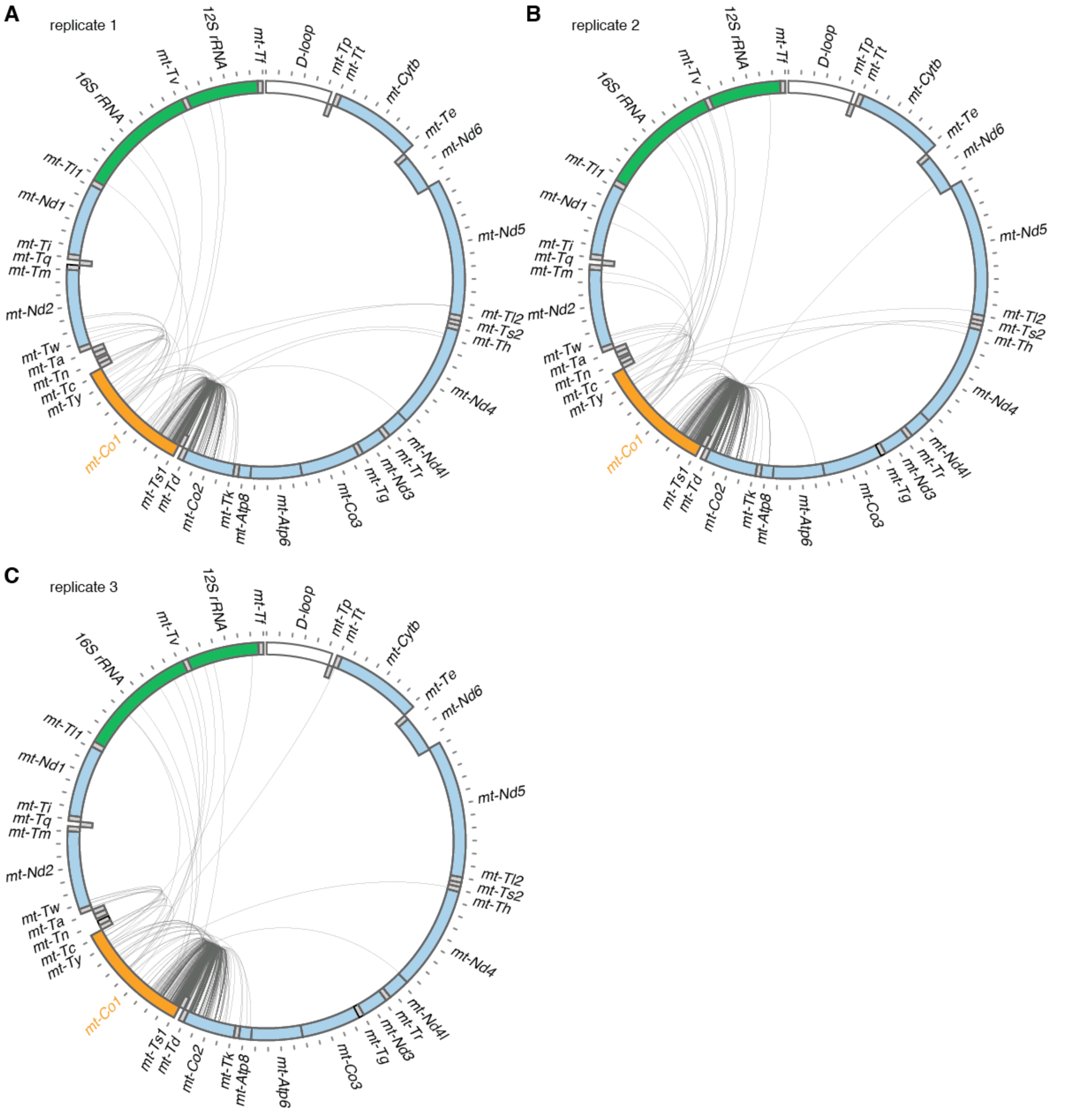

Supplementary Figure 1. Reproducibility of circularized RNA sequencing libraries and analyses. Partially processed transcripts containing *mt-Co1* from three independent libraries, prepared from three independent *Mrpp3* knock out mice, are shown as an example. Replicates 1 (A), 2 (B), and 3 (C) are shown with reads mapping between *mt-Co1* and other mitochondrial RNAs illustrated by lines between the 5′ and 3′ nucleotide positions. The locations of genes are shown in a schematic, where heavy strand genes are shown in the outer ring and light strand genes in the inner ring. Genes encoding rRNAs are shown in green, mRNAs are shown in light blue, tRNAs in grey, the D-loop in white, and the mRNA of interest, *mt-Co1*, is shown in orange.

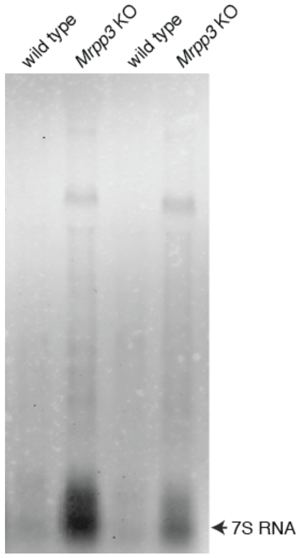

Supplementary Figure 2. Levels of the 7S RNA are increased in *Mrpp3* knock out mice. A northern blot of heart RNA from wild-type and *Mrpp3* knock out mice was hybridized with a probe specific for the 7S RNA.

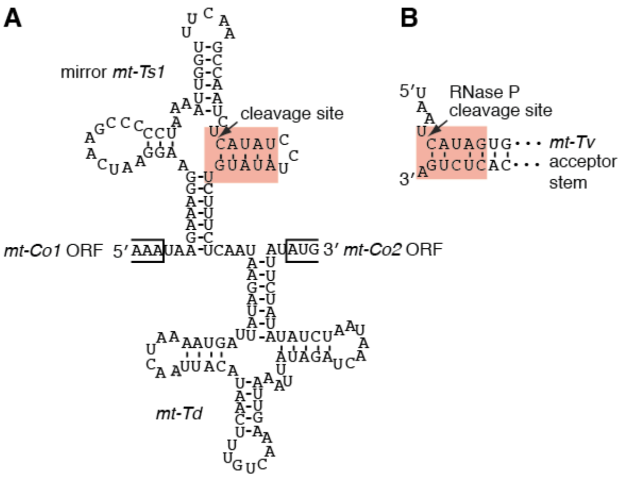

Supplementary Figure 3. (A) The predicted secondary structure for the RNA from which the *mt-Co1* 3′-UTR is derived. The cleavage site required to generate the final 3′-UTR is indicated by an arrow. (B) A section of the *mt-Tv* acceptor stem is shown to illustrate the similarity between a canonical RNase P cleavage site and the site that is cleaved to generate the *Co1* 3′-UTR.

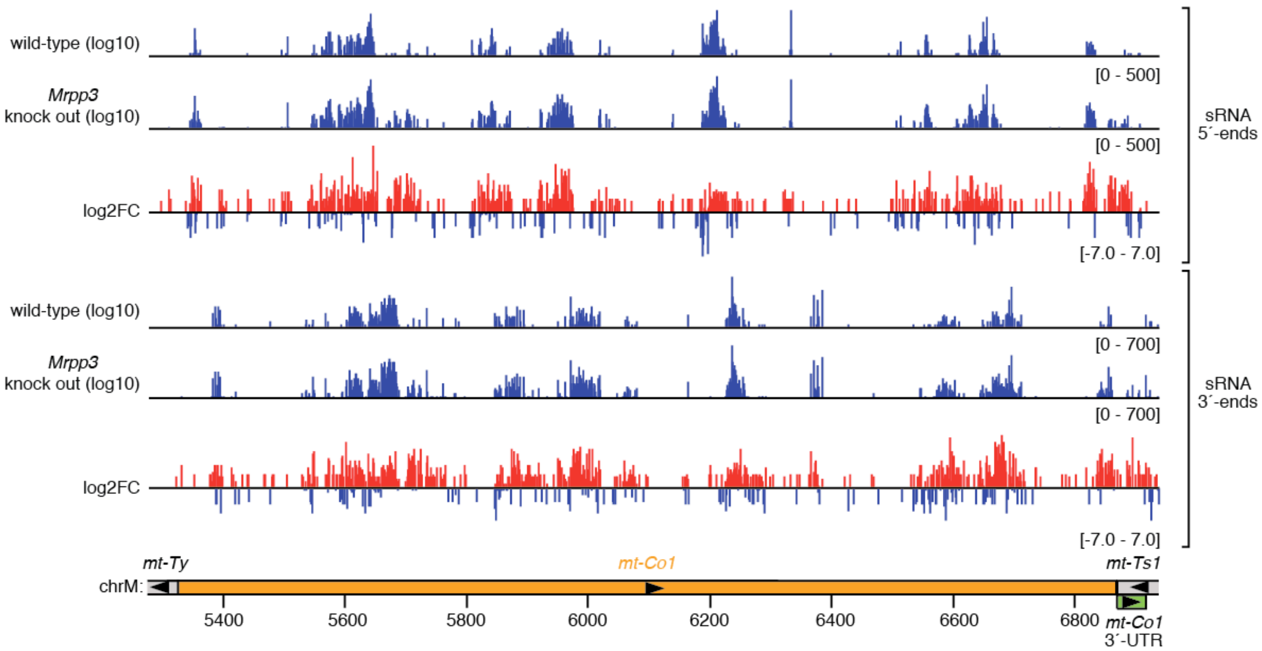

Supplementary Figure 4. Locations of sRNA 5′ and 3′ ends within *mt-Co1* and the fold change between wild-type and *Mrpp3* knockout mitochondrial circularized RNA libraries, upon normalization to reads mapping to the whole genome.

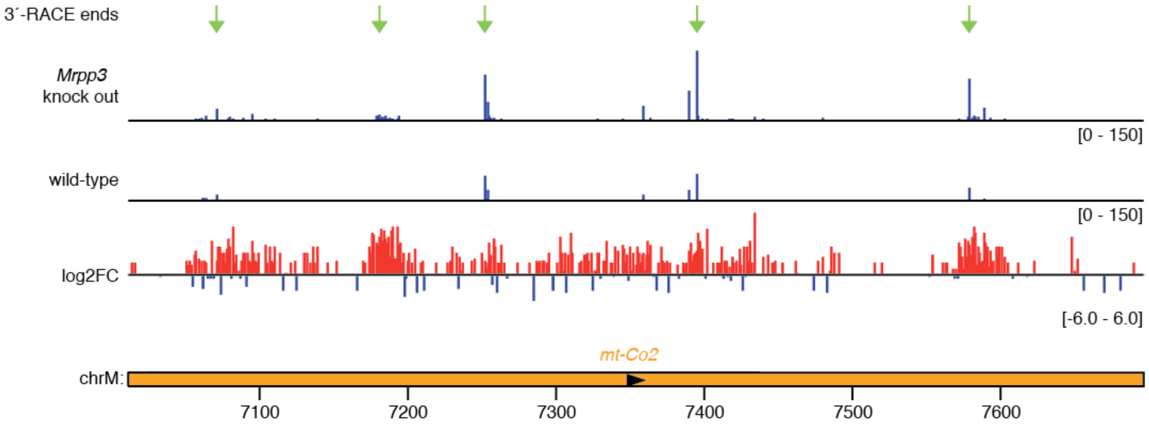

Supplementary Figure 5. Correspondence between small RNA degradation products in *mt-Co2* and partially processed transcripts containing *mt-Co1* that extend into this region identified by 3′-RACE. Locations of sRNA 3′ ends within *mt-Co2* and the fold change between wild-type and *Mrpp3* knockout mitochondrial circularized RNA libraries are shown. Locations of RNA ends identified by 3′ RACE are indicated by green arrows.

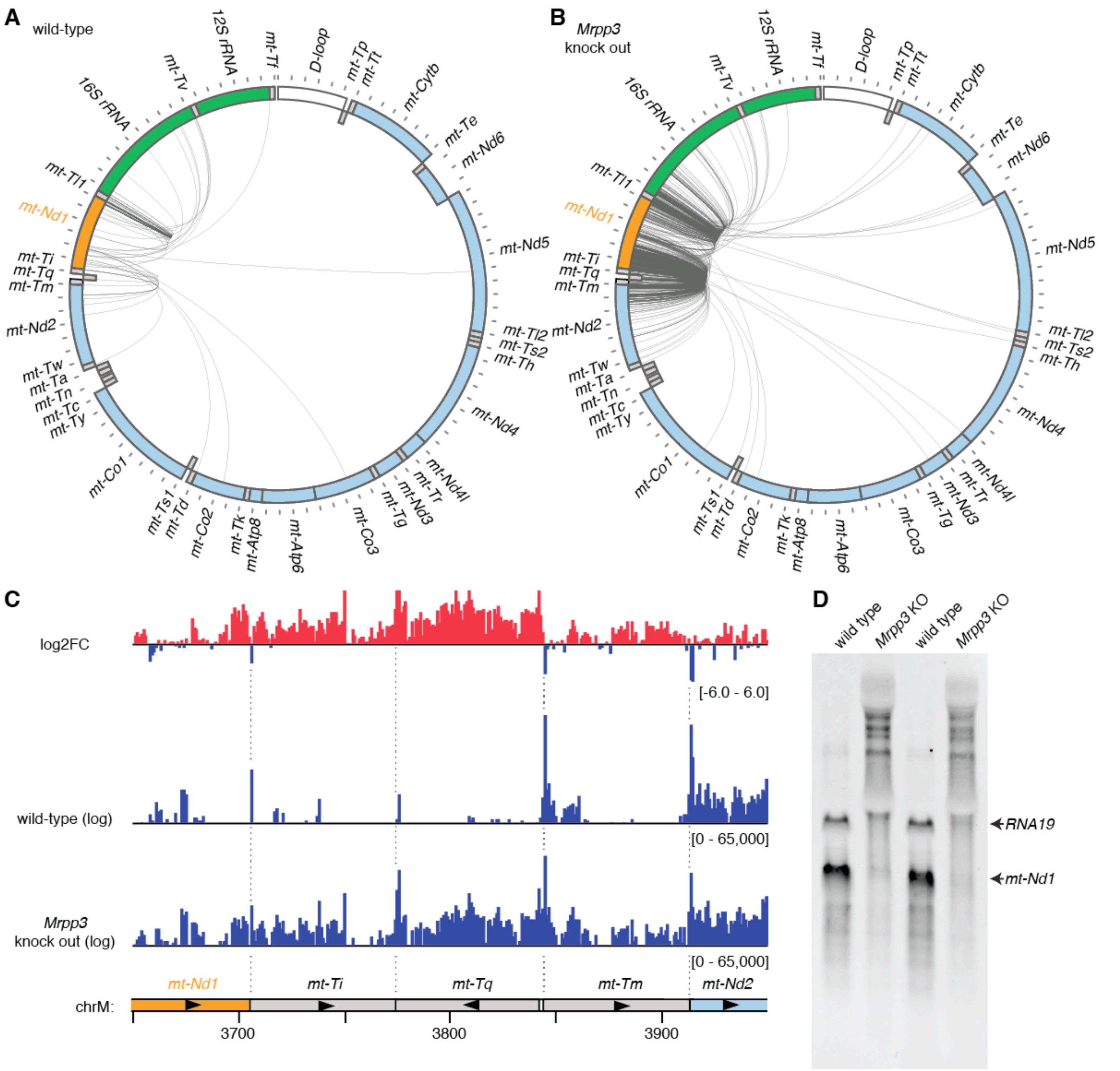

Supplementary Figure 6. Analysis of partially processed transcripts containing *mt-Nd1*. (A) Circularized RNA sequencing of wild-type mouse mitochondrial RNA. Reads mapping between *mt-Nd1* and other mitochondrial RNAs are illustrated by lines between the 5′ and 3′ nucleotide positions. The locations of genes are shown in a schematic, where heavy strand genes are shown in the outer ring and light strand genes in the inner ring. Genes encoding rRNAs are shown in green, mRNAs are shown in light blue, tRNAs in grey, the D-loop in white, and the mRNA of interest, *mt-Nd1*, is shown in orange. (B) Circularized RNA sequencing of mitochondrial RNA from *Mrpp3* knock out mice. (C) PARE data illustrating the altered 5′ and 3′ processing of *mt-Ti*, *mt-Tq* and *mt-Tm* in the absence of MRPP3. (D) Northern blotting of heart RNA from wild-type and *Mrpp3* knock out mice using a probe specific for *mt-Nd1*.

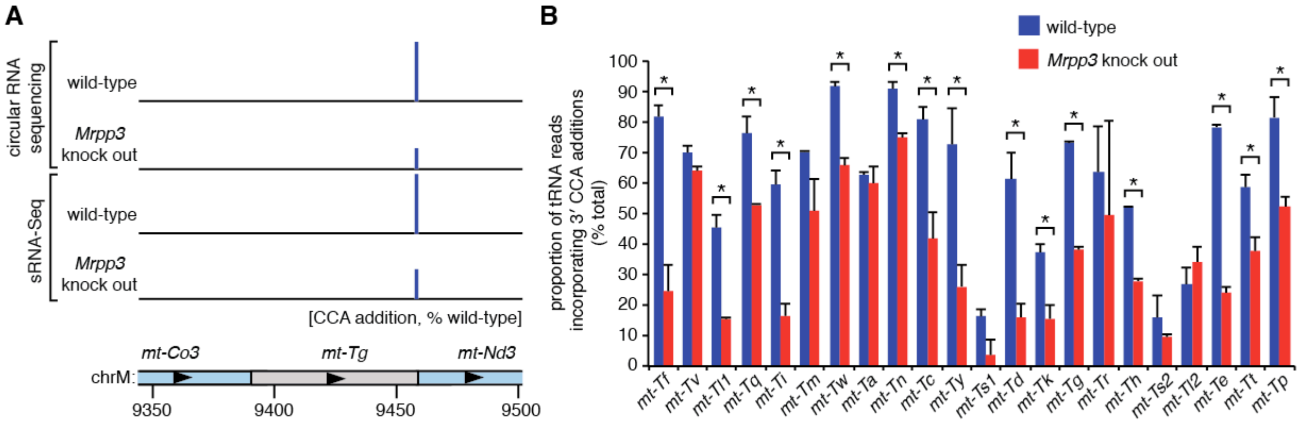

Supplementary Figure 7. Analysis of CCA addition to the 3′ ends of mitochondrial tRNAs. (A) Comparison of circularized RNA sequencing and classical small RNA (sRNA) sequencing of tRNA CCA addition in wild-type and *Mrpp3* knockout mouse mitochondria at the *mt-Tg* locus. (B) CCA addition is reduced in reads derived from *Mrpp3* knockout mouse mitochondrial tRNAs. The proportion of tRNA reads incorporating 3′ CCA additions were significantly decreased upon loss of MRPP3 (Student's *t*-test, $p \leq 0.05$, indicated by an asterisk) for the following tRNAs: *mt-Tf*, *mt-Tl1*, *mt-Tq*, *mt-Ti*, *mt-Tw*, *mt-Tn*, *mt-Tc*, *mt-Ty*, *mt-Td*, *mt-Tk*, *mt-Tg*, *mt-Th*, *mt-Te*, *mt-Tt*, and *mt-Tp*.
